# The Treasures Locked up

**Published:** 1865-11

**Authors:** 


### THE TREASURES LOCKED UP.

The Surgeon General has forbidden all medical officers giving information of officially recorded facts of the medical and surgical experiences of the war. All outside of official positions must fold their hands and wait for the scientific feast preparing in the official kitchen at Washington, after the maxim, probably, that *too many cooks spoil the broth*. This does very well for those inside, but we understand there are some outside experts who claim that they might be let into the labor, with no damage to those now at work at the vast amount of raw material, and with infinite advantage to the great public, at whose expense these records have been made and preserved. If this has come to be the general policy at Washington, we may expect soon to hear that Horace Greeley is shut out from access to the archives in his department of history.

Dr. Brandt, a distinguished Scandinavian Physician, who has recently been traveling in this country, in a communication to one of the journals of his own country, speaks highly of our hospitals and the prominent members of the profession in America. Of Prof. Brainard he says: "He stands foremost in the country, and is remarkable for his originality as a Surgeon, and as a Lecturer, he is clear, comprehensive, and practical."

Obituary.—Died, of Dysentery, Josiah Lee, M. D., at Fairmount, Vermillion Co., Oct. 13, 1865.

